# Supplementary figures and images for: Health care professionals’ experiences of screening immigrant mothers for postpartum depression–a qualitative systematic review
Source: PLoS One. 2022 Jul 14;17(7):e0271318. doi: 10.1371/journal.pone.0271318 (PMC9282607; doi:10.1371/journal.pone.0271318)

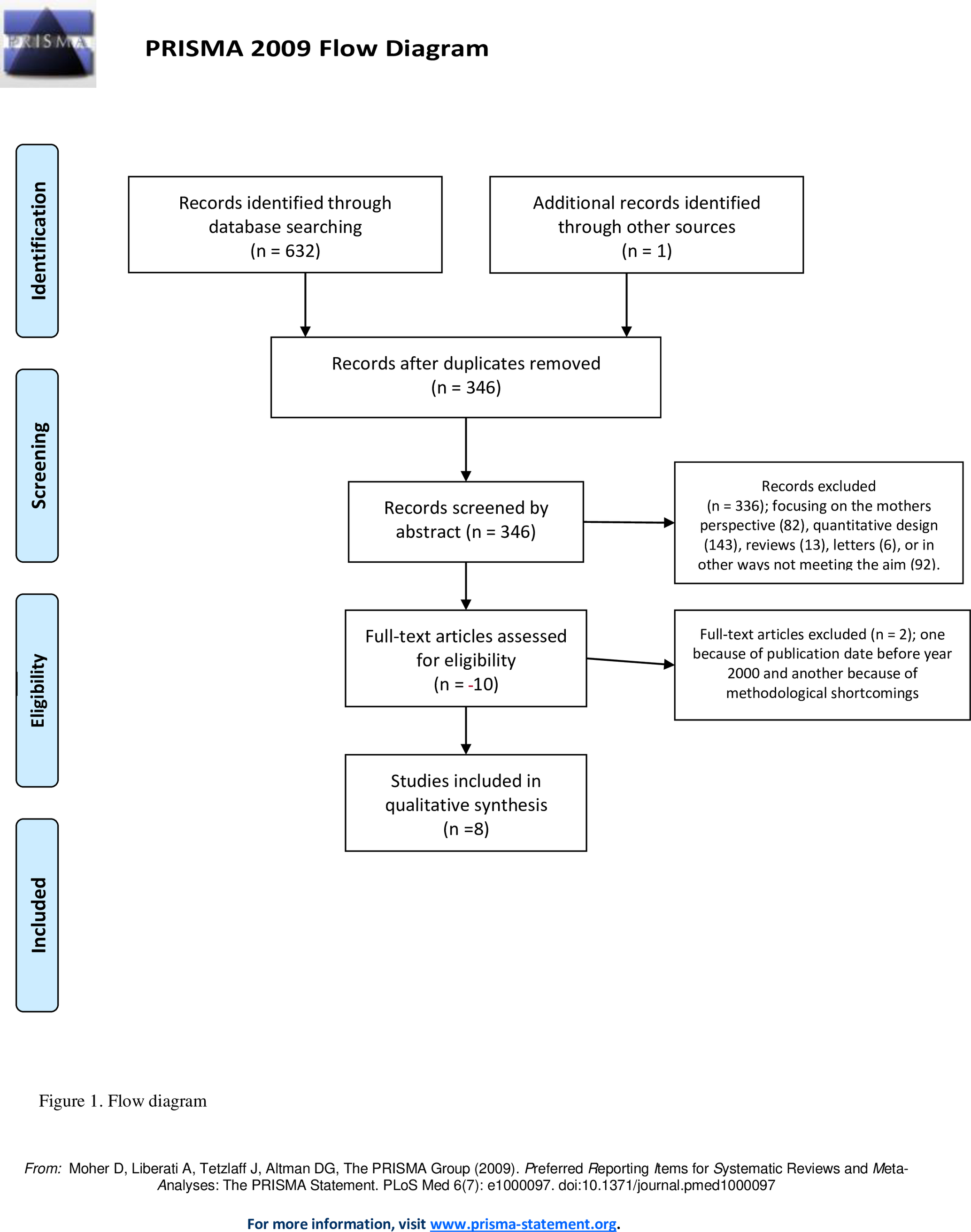

Supplement: S1 Fig — (TIF) [file pone.0271318.s002.tif]

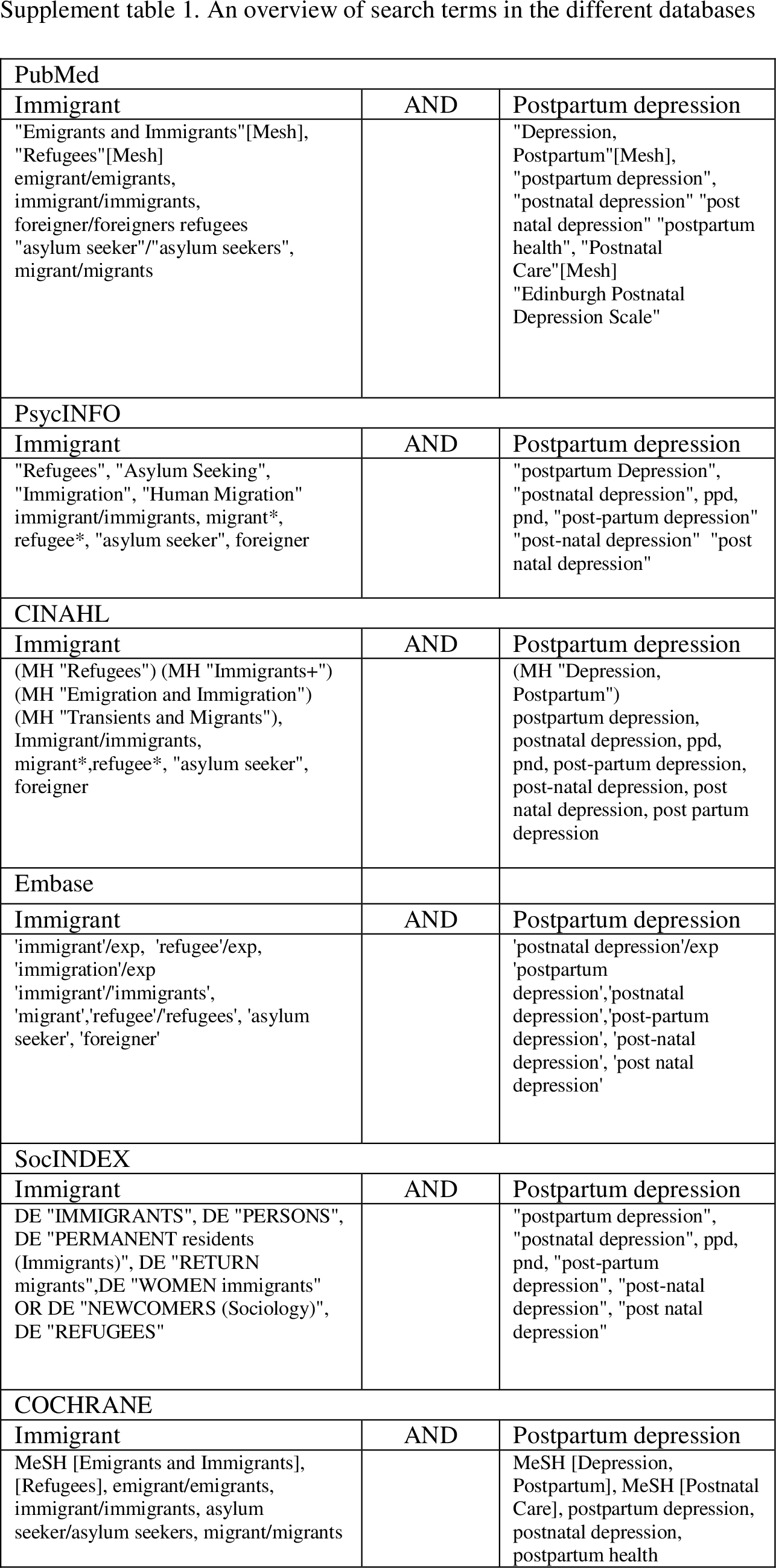

Supplement: S1 Table — (TIF) [file pone.0271318.s003.tif]

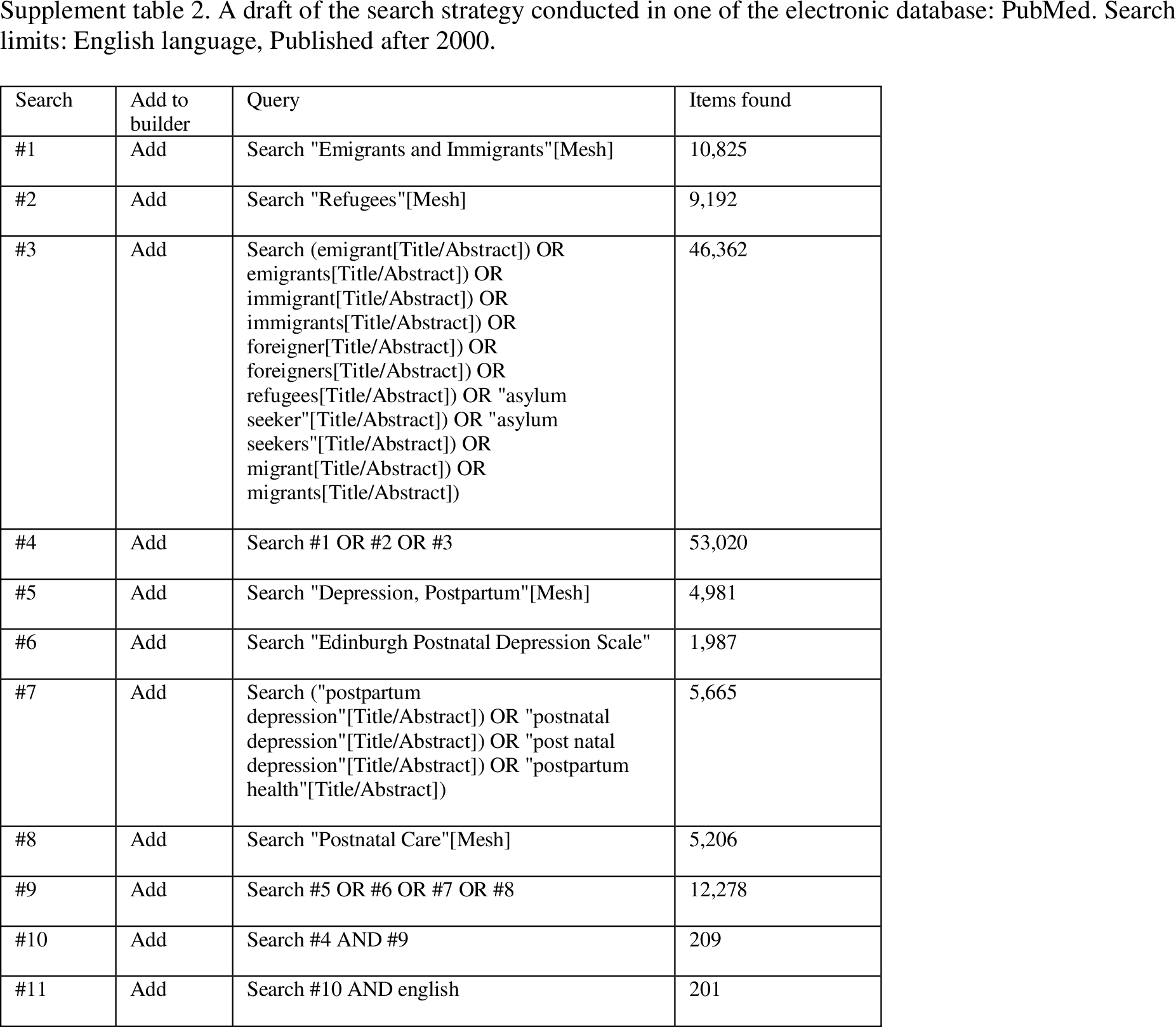

Supplement: S2 Table — (TIF) [file pone.0271318.s004.tif]
