# Supplementary material for: Health care professionals’ experiences of screening immigrant mothers for postpartum depression–a qualitative systematic review
Source: PLoS One. 2022 Jul 14;17(7):e0271318. doi: 10.1371/journal.pone.0271318 (PMC9282607; doi:10.1371/journal.pone.0271318)
Supplement: Attachment — Submitted filename: Skoog et al Response to Reviewers.docx [file pone.0271318.s006.docx]

Dear senior editor of PLoS ONE and Reviewers,

Thank you for reviewing our manuscript entitled “Health care professionals experiences of screening immigrant mothers for postpartum depression – a meta-synthesis” and your constructive suggestions for improvement.

The manuscript has been revised and the following changes has been made in response to reviewers:

| Reviewer 1 - comments |  |
| --- | --- |
| Considering method, the section on search strategy and screening, as well as the section on data extraction and synthesis, could be shorter. Table 1 and Table 2 could be presented as supplementary materials. | Thank you for this constructive comment. The sections on search strategy and screening have been shortened, while the section on data extraction and analysis are presented in a table. Table 1 and Table 2 are as suggested presented as supplementary materials. |
|  |  |
| Search outcomes: One article received CASP scores below 6. What was this article about? Do the results differ from the eight other studies? | The article in question was about postpartum depression screening in Yemeni and Punjabi Immigrant Women focusing on different examples of challenges connected to PPD screening. The results do not differ from the other studies. |
|  |  |
| Description of the included studies: Table 3 could be shorter. For example, I think it is not necessary to present titles and all authors (first author et al) could be adequate. | Thank you for this suggestion. The table has been shortened accordingly. |
|  |  |
| Discussion: A few words on PPD management in these women could be added. | Thank you for this valuable suggestion. A section has been added on HCPs experiences of PPD management in immigrant mothers. |
|  |  |
| Abstract: Introduction add HCPs after health care professionals. | Thank you noticing this. HCPs has been added. |
|  |  |
| Discussion: First sentence I think it is eight and not seven studies. | This has been changed accordingly |
|  |  |
|  |  |
| Reviewer 2 - comments |  |
| Some discussions on other screening tools of depressive symptoms should come in the discussion | Thank you for this suggestion. Examples of other screening tools for postpartum depression are now presented in the introduction. |
| I would suggest the authors should present different phases of data extraction and synthesis in a graphical way. | Thank you for your suggestion. The phases of data extraction and synthesis are presented in a table. |
| The authors should state the implications of the findings in the context of LMIC countries. | Thank you for pointing this out. A section has been added in the discussion on management of PPD where LMIC countries are mentioned. |
|  |  |
| Senior editor- comments |  |
|  |  |
| Please ensure that your manuscript meets PLoS ONE’s style requirements, including those for file naming. | Thank you for clarifying this. The supporting file is saved as S1_fig.pdf and “meta-synthesis” has been replaced by “systematic review” in the title. |
| Please ensure that you have addressed all items recommended in the Prisma checklist including identifying the study as a systematic review in the title. | Thank you for pointing this out. The checklist has been updated and “systematic review” has replaced “meta-synthesis” in the title. |
| Thank you for stating the following financial disclosure “No. The funders had no role in the study design, data collection and analysis, decision to publish, or preparation of the manuscript”.  At this time, please address the following queries:   a) Please clarify the sources of funding (financial or material support) for your study. List the grants or organizations that supported your study, including funding received from your institution. b) b) State what role the funders took in the study. If the funders had no role in your study, please state: “The funders had no role in study design, data collection and analysis, decision to publish, or preparation of the manuscript.” c) If any authors received a salary from any of your funders, please state which authors and which funders. d) If you did not receive any funding for this study, please state: “The authors received no specific funding for this work.   Please include your amended statements within your cover letter; we will change the online submission form on your behalf. | Thank you for clarifying this. The financial disclosure has been changed into “The authors received no specific funding for this work”, which also has been amended in the cover letter. |
| In your Data Availability statement, you have not specified where the minimal data set underlying the results described in your manuscript can be found. PLOS defines a study's minimal data set as the underlying data used to reach the conclusions drawn in the manuscript and any additional data required to replicate the reported study findings in their entirety. All PLOS journals require that the minimal data set be made fully available. Upon re-submitting your revised manuscript, please upload your study’s minimal underlying data set as either Supporting Information files or to a stable, public repository and include the relevant URLs, DOIs, or accession numbers within your revised cover letter. | Thank you for pointing this out. The minimal data set underlying the results is available at the repository Zenodo.  Our statement is now the following:  **“Data Availability:** The dataset used in this study is available here: <https://zenodo.org/record/5774074#.YbTVYC9rJQI>  DOI 10.5281/zenodo.5774073. |
| Please note that in order to use the direct billing option the corresponding author must be affiliated with the chosen institute. Please either amend your manuscript to change the affiliation or corresponding author, or email us at plosone@plos.org with a request to remove this option. | The corresponding author is affiliated with the chosen institute. |
| Please include captions for your Supporting Information files at the end of your manuscript, and update any in-text citations to match accordingly. | Thank you for clarifying this. Captions for the supporting information files are changed. |
| Please remove all personal information, ensure that the data shared are in accordance with participant consent, and re-upload a fully anonymized data set. Note: spreadsheet columns with personal information must be removed and not hidden as all hidden columns will appear in the published file. | Thank you for pointing this out. Since this is a systematic review there is no personal information. |

Thank you once again for reviewing the manuscript and for you constructive comments for improvement. We hope you are satisfied with the changes.

Kind regards,

On behalf of the authors
Malin Skoog
RN, PhD student
Lund University Sweden
malin.skoog@med.lu.se
