# Supplementary material for: Health care professionals’ experiences of screening immigrant mothers for postpartum depression–a qualitative systematic review
Source: PLoS One. 2022 Jul 14;17(7):e0271318. doi: 10.1371/journal.pone.0271318 (PMC9282607; doi:10.1371/journal.pone.0271318)
Supplement: Attachment — Submitted filename: Response to Reviewers 220609.docx [file pone.0271318.s007.docx]

Dear senior editor of PLoS ONE and Reviewers,

Thank you for reviewing our manuscript entitled “Health care professionals experiences of screening immigrant mothers for postpartum depression – a meta-synthesis” and your constructive suggestions for improvement.

The manuscript has been revised and the following changes has been made:

| Reviewer 1 - comments |  |
| --- | --- |
| Reviewer 2 suggested some discussions on other screening tools of depressive symptoms in the discussion. The authors presented this point in the introduction. I think it could be in the discussion, as reviewer 2 suggested. | Thank you for noticing this. The section on other screening tools of depressive symptoms have been moved to the discussion. |
|  |  |
| Reviewer 3 - comments |  |
| Suggest stating aim more clearly in the abstract. In addition, the aim needs to be clearly articulated either prior to or at the start of methods. What is written there is more an implied aim for the review. | Thank you for your constructive comment. The aim has been clarified in both abstract and introduction. The abstract now reads “The aim of this study was to synthesize health care professionals (HCPs) experiences of identifying signs of postpartum depression and performing screening on immigrant mothers, since previous research suggested that this might be challenging” and the introduction “The aim of this study was therefore to synthesize health care professionals’ experiences of identifying signs of PPD and screen immigrant mothers for the condition”. |
| As an overall point I wondered why the review had not included – antenatal or perinatal depression. Often screening using the EPDS etc is routine in pregnancy and many HCPs have commented on the challenges they experience. This stood out for me even more when I noted that at least three of the papers included in the review actually focus on the perceptions of HCPs who work in antenatal settings -for example the research by Nithianandan; Stapleton and Willey. I would suggest that the review should either have the broader focus or at least the intention to include studies of HCPs working antenatally with women as well as following birth. This could also be addressed in the limitations. | Thank you for noticing this. A section was added under limitation explaining that ”Because of a limited number of studies found focusing HCPs experiences of performing screening on immigrant mothers we chose to include three studies focusing on antenatal or perinatal screening instead of solely postpartum. |
| In the Introduction I noted that a number of points particularly related to prevalence of PPD are referenced to quite old papers eg refs 3,4,8,9,13 | Thank you for pointing this out. Reference 3 and 4 has been replaced and reference 8 and 9 has been removed. Reference 13 was kept since it is an important reference for arguing the relevance of the paper. |
| Suggest all these scales need referencing here in introduction “There are specific scales developed to screen for symptoms of depression in the peripartum and postpartum period; the Edinburgh Postpartum Depression Scale (EPDS), the Postpartum Depression Screening Scale and the  Pregnancy Risk Questionnaire. | Thank you noticing this. References had been added for the different scales. |
| Screening also appears to be acceptable to women – Kingston is a good reference for that particularly in primary care. | Thank you for pointing this out. The suggested reference Kingston was not retrieved, and another reference was therefore used. |
| I suggest that each time indigenous is used it should eb with a capital ‘I” but then I think that you may be referring to the dominant population group in a country. This needs clarifying please. | Thank you for pointing this out. The term indigenous has been clarified. |
| Results. I think you have done an excellent job in identifying and articulating the translation of the key themes  I had wondered about the title of the first sub theme – referring to being a good friend… I think it is a little misleading in terms of the practice of the HCP because they correct themselves and do not emphasise becoming friends rather the quote says “…but that we have a good relationship. That they feel they are liked and that I listen to them. I…”  In the second sub theme I am reading here the importance of continuity and seeing women overt time to recognise the change in their presentation or demeanour. I think you do address this in the discussion but it is a very important point. | Thank you so much for your positive comment. The title of the first subcategory has been changed to; “I was about to say become friends, but it’s the wrong word” to avoid misleading the reader. |
| The discussion covers important issues and addresses the findings  I wondered if more could be said about taking a different approach to screening whereby the EPDS or other tool is used as the basis of a conversation rather than just the pen and paper exercise. But this more conversational approach is probably much more relevant for the psychosocial questions that may accompany the screening using a tool. | Thank you for raising this interesting point. This is an important question, but we feel that it belongs in another context where it can be given the space it deserves. |
|  |  |

Thank you once again for reviewing the manuscript and for your constructive comments for improvement. We hope you are satisfied with the changes.

Kind regards,

On behalf of the authors

Malin Skoog
RN, PhD
Lund University Sweden
malin.skoog@med.lu.se
