# Supplementary material for: Health care professionals’ experiences of screening immigrant mothers for postpartum depression–a qualitative systematic review
Source: PLoS One. 2022 Jul 14;17(7):e0271318. doi: 10.1371/journal.pone.0271318 (PMC9282607; doi:10.1371/journal.pone.0271318)
Supplement: Attachment — Submitted filename: Skoog et al, Response to Editor 220627.docx [file pone.0271318.s008.docx]

Dear editor of PLoS ONE,

Thank you for reviewing our manuscript entitled “Health care professionals experiences of screening immigrant mothers for postpartum depression – a qualitative systematic review” and your constructive suggestions for improvement.

The manuscript has been revised and the following changes has been made:

| Editor - comments |  |
| --- | --- |
| The authors should revise the title of their manuscript to describe the study as a "qualitative systematic review" | Thank you for constructive comment. The word qualitative has been added to the title. |
| The authors should cite the ENTREQ reporting guidelines in the Methods section, along with completing the ENTREQ checklist. | Thank you for pointing this out. The ENTREQ reporting guidelines was added in the Methods section and the checklist is attached. |
| In Table 3 where the word "women" should replace "woman", or else indicate this was the verbatim quote using. | Thank you for noticing this. The word “woman” was replaced with the word “women” in Table 3. |
| Please review your reference list to ensure that it is complete and correct. | Thank you for pointing this out. One reference had gone missing (5.Cameron EE, Sedov ID, Tomfohr-Madsen LM. Prevalence of paternal depression in pregnancy and the postpartum: An updated meta-analysis. Journal of Affective Disorders. 2016; 206:189-203. Doi: 10.1016/j.jad.2016.07.044 PMID:27475890), and one reference was added (32.Tong A, Flemming K, McInnes E, Oliver S, Craig J. Enhancing transparency in reporting the synthesis of qualitative research: ENTREQ. BMC Medical Research Methodology 2012 12:181. doi:10.1186/1471-2288-12-181 PMID: 23185978) |
| Please upload your figure files to the Preflight Analysis and Conversion Engine (PACE) digital diagnostic tool, https://pacev2.apexcovantage.com/. | Thank you for pointing this out. The figure files were uploaded into PACE. |

Thank you once again for reviewing the manuscript and for your constructive comments for improvement. We hope you are satisfied with the changes.

Kind regards,

On behalf of the authors

Malin Skoog
RN, PhD
Lund University Sweden
malin.skoog@med.lu.se
